# Multimode Optical Interconnects on Silicon Interposer Enable Confidential Hardware-to-Hardware Communication

**DOI:** 10.3390/s23136076

**Published:** 2023-07-01

**Authors:** Qian Zhang, Sujay Charania, Stefan Rothe, Nektarios Koukourakis, Niels Neumann, Dirk Plettemeier, Juergen W. Czarske

**Affiliations:** 1 Laboratory of Measurement and Sensor System Technique, Faculty of Electrical and Computer Engineering, TU Dresden, 01069 Dresden, Germany; stefan.rothe@tu-dresden.de (S.R.); nektarios.koukourakis@tu-dresden.de (N.K.); juergen.czarske@tu-dresden.de (J.W.C.); 2 Chair of Radio Frequency and Photonics Engineering, Faculty of Electrical and Computer Engineering, TU Dresden, 01069 Dresden, Germany; dirk.plettemeier@tu-dresden.de; 3 Institute for Electrical Information Technology, TU Clausthal, 38678 Clausthal-Zellerfeld, Germany; niels.neumann@tu-clausthal.de; 4 Institute of Applied Physics, School of Science, TU Dresden, 01069 Dresden, Germany

**Keywords:** silicon interposer, physical layer security, multimode optical interconnect, through-silicon via, wavefront shaping, deep neural network

## Abstract

Following Moore’s law, the density of integrated circuits is increasing in all dimensions, for instance, in 3D stacked chip networks. Amongst other electro-optic solutions, multimode optical interconnects on a silicon interposer promise to enable high throughput for modern hardware platforms in a restricted space. Such integrated architectures require confidential communication between multiple chips as a key factor for high-performance infrastructures in the 5G era and beyond. Physical layer security is an approach providing information theoretic security among network participants, exploiting the uniqueness of the data channel. We experimentally project orthogonal and non-orthogonal symbols through 380 μm long multimode on-chip interconnects by wavefront shaping. These interconnects are investigated for their uniqueness by repeating these experiments across multiple channels and samples. We show that the detected speckle patterns resulting from modal crosstalk can be recognized by training a deep neural network, which is used to transform these patterns into a corresponding readable output. The results showcase the feasibility of applying physical layer security to multimode interconnects on silicon interposers for confidential optical 3D chip networks.

## 1. Introduction

Powerful microelectronic systems represent a cornerstone for digital transformation [1]. On this path, emerging cyber-physical systems (CPSs) are considered an advanced approach, especially for the Internet of Things, as they combine cyber and physical components into an embedded realization [2]. For such systems, chips from different technologies have to be integrated and a large amount of information has to be exchanged with each other. Three-dimensional integration on interposers provides one promising solution for a photonic CPS to tackle these challenges [3]. On interposers, high-speed links between chips are established with multimode optical interconnects. These can be horizontal with integrated planar waveguides or vertical with optical through-silicon vias (OTSVs). This work focuses on OTSVs, which are the shortest possible connections on an interposer.

**Figure 1:**
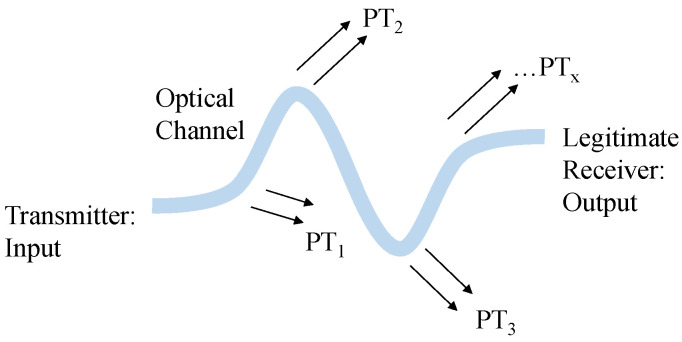
Data transmission in optical channels in the presence of potential eavesdroppers. Abbreviation: PT, potential threat.

However, the multitude of innovative applications in the digital domain including CPSs attracts novel types of cyber-attacks [4,5]. Especially for optical communication applications, eavesdroppers can intercept data transmitted over optical or electrical media without being revealed. In optical communication, evanescent coupling in waveguides allows an attacker to operate passively without channel penetration [6,7,8,9]. The potential threats could be data theft via eavesdropping, communication disruption, etc. Potential threats arise particularly in areas with small bend radii, where the guided waves could be radiated (see Figure 1).

Classical approaches to prevent eavesdropping, such as cryptography, have some severe trade-offs. Among others, there are asymmetric keys, such as RSA (public-key cryptosystem) codes, which rely on the computational complexity of the prime factorization of large numbers. However, with the advent of a quantum computer [10], it could be cracked. Furthermore, it could be considered weak in energy consumption and latency [11]. Physical unclonable functions (PUF) could eliminate these disadvantages since no mathematical computation is required to create a key [12,13]. Instead, random processes or unique tokens are exploited to generate, for instance, an optical PUF which enables secure authentication [14]. However, applications of PUFs are struggling with noisy responses or sensitivity to the environment, which makes the generated key unreliable. There are already investigations to address these challenges by increasing the overhead through error corrections [15].

Physical layer security (PLS) approaches help to tackle these issues. It was shown in Wyner’s seminal work [16] that there is a class of channel codes, namely wiretap codes [17], that provide secure authentication or confidentiality by using the physical properties of the channel without relying on a key exchange. In addition to the noisy channels investigated there, this approach is also prominent in wireless networks [18,19] that exploit, amongst other things, fading [20].

The development of adaptive optical elements, such as a spatial light modulator or digital micromirror device (DMD), enables control of the the propagation of light through disordered media [21,22,23]. This allows us to overcome the complex light transport of multimode fibers (MMFs) and exploit them for PLS [24,25,26]. Light propagation through MMF is characterized by phenomena such as crosstalk or mode-dependent losses, which are not predictable in real applications. However, they can be measured and controlled by adaptive optics and wavefront shaping, making the MMF a suitable candidate for employing PLS. Although stability issues due to environmental changes result in strong challenges for the application of PLS in MMF, it is considered a promising approach [9] because PLS is based on classic light. Contrary to non-classic approaches, such as quantum key distribution [27], PLS has a high level of integrability into communication environments, including amplifiers or repeaters.

In this paper, we investigate the feasibility of PLS for multimode optical waveguides, fabricated directly on silicon interposers [28]. We focus on OTSVs, which should provide two crucial properties to enable compatibility for PLS. First, the OTSV should behave as a linear time-invariant system. Unlike MMFs, which show severe time variance due to environmental influence [29], we hypothesize that short optical paths in an OTSV lead to drastically enhanced robustness. Secondly, each and every OTSV should be unique. This means the inherent process variations, such as random side-wall roughness, should lead to individual channel responses for different realizations on one interconnect as well as between different samples. Within an experiment, we investigate how sufficient modal crosstalk develops along the short optical paths of an OTSV. To explore the essential characteristics of linearity and uniqueness, we project different classes of optical functions on the OTSV input using a DMD and analyze the corresponding patterns at the output using both correlation and a neural network. Our work is structured as described below. First, we introduce the fabrication of the OTSVs on silicon interposers and their application into optical embedded systems. Then we offer explanations of the experimental setup and the characterization procedure. Finally, we discuss the results and give an outlook for further applications.

## 2. Fabrication of OTSVs on Silicon Interposer and Its Performance

**Figure 2:**
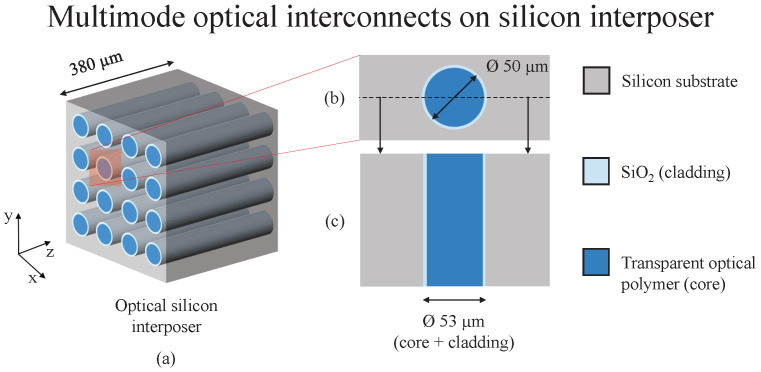
(**a**) Optical silicon interposer with OTSVs. (**b**) one OTSV (top view); (**c**) one OTSV (cross-section).

An interposer is an intermediate routing interface between two chiplets. It enables a higher interconnect density and, hence, 2.5D and 3D chip integration [30,31] are possible. Through-silicon via (TSV), as the name suggests, is a through-connection between the top layer and the bottom layer of the silicon interposer. Figure 2 illustrates the structure of OTSVs within a silicon interposer. There are two kinds of TSVs possible, i.e., electrical and optical. The TSV fabrication process remains similar for both types of TSVs. The fabrication starts with lithography on a 380 μm (±5 μm—total wafer thickness variation) silicon wafer, where a predefined circular opening is achieved in the photoresist layer. A vertical silicon etching is followed after that by an anisotropic (BOSCH) dry etch process. A high-aspect-ratio (from 8:1 up to ≈30:1) etching is carried out, making a through-opening in the silicon interposer [32]. After that, approx. 1.5 μm of thick silicon oxide is thermally grown on the silicon interposer. This step has a dual purpose. For electrical TSVs, the silicon oxide functions as an insulator, and, for the OTSVs, the silicon oxide functions as a cladding layer (with a refractive index of $n_{clad}\approx 1.45\pm 0.01$ for the VIS-IR region). At this point, the processing for OTSVs differs from the electrical TSVs. These TSVs can be turned into OTSVs by filling them with optically transparent polymer that has a higher refractive index $n_{core}$ than $n_{clad}$. For this application, the optical polymer Ormocore was used as the core material, which has a refractive index ≈1.55±0.01 for the VIS-IR region. Analogous to a multimode fiber, the diameter of the OTSV is kept at 53 µm (a 50 µm diameter as waveguide core region and 1.5 µm surrounding the region as cladding). Modeling such a short-distance OTSV on the silicon interposer indicates that the theoretical bandwidth for such a waveguide is in THz range [33].

As shown in [34], these OTSVs can be extended with horizontal waveguides, producing an end-to-end optical waveguide network. With the help of optical coupling elements, such as micro-mirrors, beam splitters, and the optical redistribution layer [35], a multimode optical interconnect network can be formed for confidential hardware-to-hardware communication. The integrated manufacturing and short distances on the interposer lead to an expected high stability and robustness of the channel. It will be shown in the later sections, that the stability and robustness of the multimode OTSVs provide an edge over off-the-shelf multimode fibers. To test the physical layer security attributes of these OTSVs, two silicon interposer samples were used. These will be referred as interposers A and B in the following sections of the paper. Both of these samples were fabricated with the same process and had more than 250 OTSVs each. These OTSVs were first tested for their optical data transmission performance. As shown in [28], these OTSVs showed an error-free data-transmission (BER $<10^{-12}$) operation of up to 40 Gbit/sec with maximum data rates up to 50 Gbit/sec (limited only by the available hardware). They rendered an extremely low loss of an order of 0.1 dB/380 μm (@850 nm—MM). With these results, such OTSVs are among the highest bandwidth-carrying and lowest-loss-posing optical interconnects on a silicon interposer.

## 3. Application Scenarios

**Figure 3:**
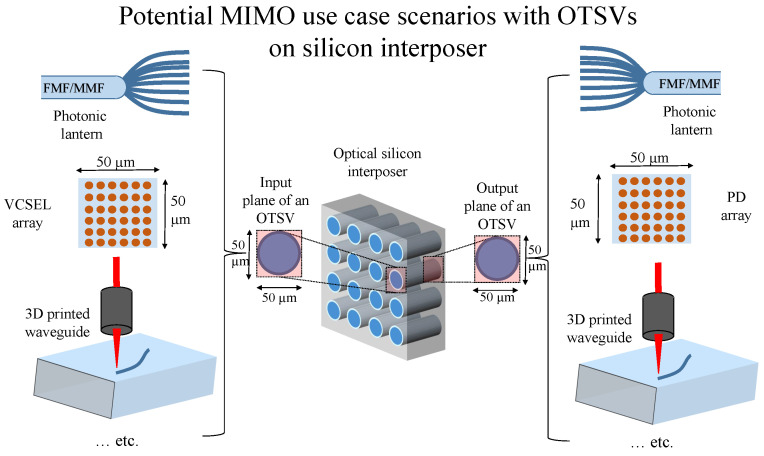
MIMO use case scenarios with optical silicon interposer.

As the techniques to fabricate photonic components improve and, with that, the performance of the photonic devices also improves, there are a few multiple-input and multiple-output (MIMO) devices that could take advantage of integration with silicon interposer. OTSVs play a vital role in many applications where a large amount of information has to be exchanged between multi-chip units. Figure 3 illustrates the combination of different technologies used in conjunction with OTSVs. The end goal is to transmit data at high speeds while simultaneously safeguarding it along the way.

The task is to project a definite image on the input plane of the OTSV by using approaches such as a photonic lantern, an array of vertical-cavity surface-emitting lasers (VCSELs), or a 3D printed multimode waveguide by two-photon lithography. Photonic lanterns do not produce light on their own but are used rather as an MM-SM-MM optical interconnection. They can be used to obtain the SM performance out of the MM devices [36,37]. The photonic lantern can be configured from MMF and also from a few-mode fiber (FMF). In order to provide a multimode input (in this case, the projection of a Latin alphabet on the OTSV input facet), the input to the photonic lantern can be tuned such that the output of the lantern projects an alphabet. The same can also be achieved through programming a VCSEL array and enabling specific elements of the VCSEL diodes. Tuning the input to the 3D printed waveguides also provides a way to deliver the required input to the MM OTSVs. Through unique multimode OTSV characteristics, the incident image undergoes mode-mixing and the resulting wavefront is collected at the output plane of the multimode OTSV. Similar to the projection of the input image, the collection of the resulting image can be accomplished by techniques such as a photonic lantern, an array of photodiodes (PDs), and a 3D printed waveguide. The ability of high-speed data transmission through these OTSVs has been previously established [28]. The next step was to examine the aforementioned OTSVs on a silicon interposer with regard to their application in physical layer security. To check the security of the data transmission, we investigate image transmission through OTSVs.

## 4. Experimental Procedure

**Figure 4:**
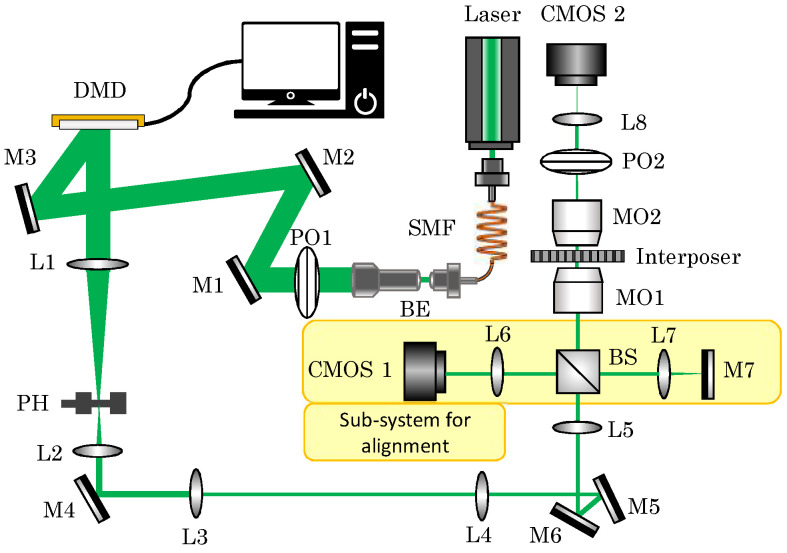
Scheme of experimental setup. Abbreviations: BE, beam expander; BS, beam splitter; L, lens; M, mirror; MMF, multimode fiber; MO, microscope objective; PH, pinhole; PO, polarizer; SMF, single mode fiber. Focal distances of lens: L1, 250 mm; L2, 50 mm; L3, 100 mm; L4, 50 mm; L5, 200 mm; L6, 200 mm; L7, 50 mm; L8, 200 mm.

In order to characterize the light propagation behavior through the interposer, an optical setup has been designed as shown in Figure 4. A laser with a wavelength of 532 nm (Diode-Pumped Solid State DPSS Laser, DJ532-10, Thorlabs) is used as the light source. The laser beam is illuminating the DMD (ViALUX V-9601), which is employed as the modulator that produces different light distributions, i.e., by means of binary Lee holograms [38]. In order to ensure the best beam profile for the experiments, the laser beam is coupled to a single-mode fiber (SMF) and collimated by a collimation package (CP). The beam is widened using a beam expander (BE) to illuminate the full screen of the DMD used. For the initial investigation, the polarization dependence is not considered; hence, a polarizer is used to filter only one polarization state from the incoming laser beam. The micromirror of the DMD rotates +12° diagonally when it is turned on. So, a down-tilt light beam is required when a reflected beam parallel to the workbench is desired. Three mirrors are employed to provide an appropriate incident angle. After illuminating the DMD screen, the beam passes through a lens (L1) performing a fast Fourier transform (FFT) with the light field. The modulated light field is then separated spatially into the respective diffraction orders. The necessary information is located in the first order, while the non-relevant information is located in the zero order. A pinhole is then placed in the Fourier domain, which allows the first diffraction order to pass but blocks the remaining information parts. Another lens (L2) with a focal distance of 100 mm is used to perform an inverse Fourier transform to transfer the spatially filtered information into the image domain. Then, a 4F system consisting of the L3 (200 mm) and L4 (100 mm) reduces the light beam size by a factor of 2. Afterward, the light field images are projected onto the input facet of the interposer through the L5 and the microscope objective MO1 (40×). Finally, an imaging system consisting of the MO2 (40×) and L8 maps the output of the interposer onto the camera CMOS2 (IDS UI-348xLE-M). Additionally, the lenses L6 and L7, the mirror M7, and the second camera CMOS1 (IDS UI-348xLE-M) constitute a sub-system for the alignment of the setup, which is introduced to ensure proper light launching conditions. The procedure of the alignment is the same as explained in prior work [39]. With the help of the DMD, the modulated light can be projected on the input facet of the OTSV with a high switching rate. An artificial intelligence method is used to analyze and evaluate the corresponding output captured by the camera.

## 5. Results and Discussion

**Figure 5:**
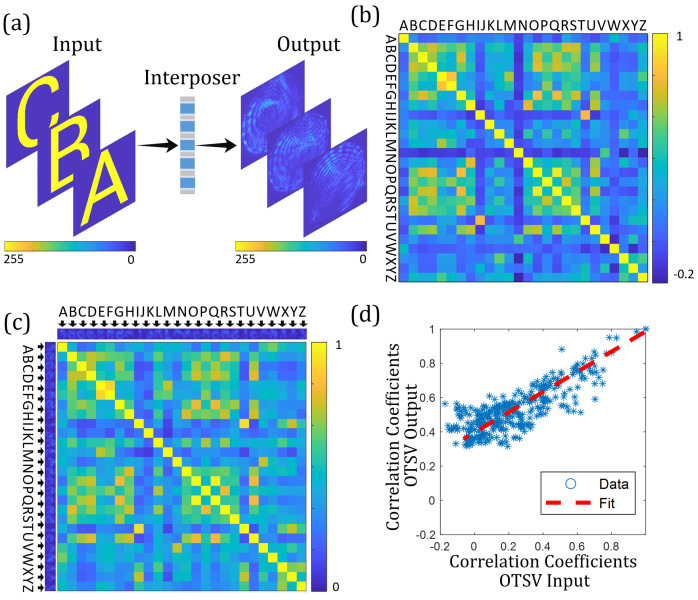
(**a**) Speckle patterns captured by the CMOS correspond to different Latin letters used as input for the OTSV. (**b**) Cross-correlation matrix of input (letters). (**c**) Cross-correlation matrix of output corresponding to the input letters. (**d**) Linear behavior between the input distributions and the output distributions. Correlation coefficients from the input base, i.e., alphabetic letters, are plotted over the correlation coefficients from output speckle patterns. The correlation coefficients are taken from matrices shown in (**b**,**c**).

**Figure 6:**
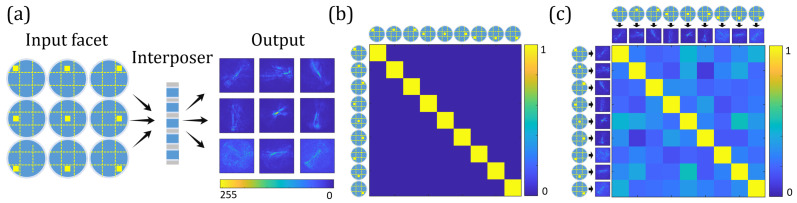
(**a**) Focal points as orthogonal input for the OTSV. The dashed line indicates that the input facet of the OTSV is divided into 9 parts. The yellow squares represent the position of focal point. Speckle patterns corresponding to the different positions of the focal point are shown in the right side of interposer. (**b**) Cross-correlation matrix of input (focal points). (**c**) Cross-correlation matrix of output speckle patterns.

To understand the light transmission properties of OTSVs, three different experimental series were carried out. In the first experiment, letters from the Latin alphabet were projected sequentially on the input side of an OTSV sample, as depicted in Figure 5a. A special feature is that the letters represent a non-orthogonal signal base. The cross-correlations of the input and the corresponding output signals are shown in Figure 5b and Figure 5c, respectively. In a second experiment, focal points are projected at locally separated positions creating an orthogonal signal base. The results are shown in Figure 6. With these two experiments, it should be shown that the respective base at the input correlates with the corresponding intensity distribution on the output side. Thus, the light transport between the input and output of an OTSV sample can be understood as a linear transformation. For this purpose, correlation studies are carried out. In a third experiment, different OTSV samples are compared with each other to investigate their uniqueness.

### 5.1. Non-Orthogonal Input Base: Alphabetic Letters

The use of alphabetic letters as a non-orthogonal test base can provide an intuitive comparison between the input and the output of OTSVs. First, we took interposer *A* and randomly selected one OTSV to project light patterns on it. Although the OTSV provides a communication channel length of only 380 μm, it induces a strong light scrambling that can be recognized at the OTSV output, as shown in Figure 5a. The projected letters can no longer be identified from the speckle patterns; however, the speckle patterns are related to each other. Concretely, the correlation properties are similar to the correlations of the input base. This can be seen in the correlation matrices shown in Figure 5b,c. Here, both the letters and the corresponding speckle patterns are correlated according to the correlation coefficient:

(1) $$\Gamma =\frac{\underset{ROI}{\begin{array}{c} \sum \end{array}}\left(I_{r}-\bar{I_{r}}\right)\left(I_{t}-\bar{I_{t}}\right)}{\sqrt{\left(\underset{ROI}{\begin{array}{c} \sum \end{array}}{\left(I_{r}-\bar{I_{r}}\right)}^{2}\right)\left(\underset{ROI}{\begin{array}{c} \sum \end{array}}{\left(I_{t}-\bar{I_{t}}\right)}^{2}\right)}},$$

where $\bar{I}$ indicates the mean value of the respective intensity distribution, and ROI indicates the region of interest. The subscript “*r*” denotes the reference intensity distribution, and “*t*” denotes the test intensity distribution. Note that all elements on the main diagonal have the value of one, as it indicates the self-correlation coefficient. The negative terms in Figure 5b represent the anti-correlation of the distribution of certain letters, such as “Z” and “N”. Comparing both matrices from Figure 5b,c, it can be found that highly correlated inputs lead to highly correlated outputs, such as “O” and “Q” or “E” and “F”. Moreover, by mapping the matrices onto each other, a linear relationship is evident, which is shown in Figure 5d. The existence of deviation can be caused by factors such as measurement noise, manufacturing tolerances of the OTSV, etc. The average value of the deviation is 19%.

### 5.2. Orthogonal Input Base: Focal Points

In order to investigate the transmission response of an orthogonal input base, the facet of the same OTSV used in the previous experiment is divided into nine zones, as shown in Figure 6a. Within the experiment, we sequentially project a focal spot on the center of the respective zone and capture the corresponding output intensity distributions. The yellow squares indicate the position and the size of the incident light beam. This configuration yields an orthogonal input base, as can be seen from the correlation matrix shown in Figure 6b. Correlating the corresponding intensity distributions captured at the output intensity, we again observe a matrix with a strong main diagonal (see Figure 6c), as can be expected from linear channel behavior. During the experiment, we ensured that the focal spots had as little overlap as possible and divided the zones accordingly. Choosing smaller zones, or focal points with a larger diameter, would lead to the development of secondary diagonal elements in the correlation matrix.

### 5.3. Comparison of Different OTSV Samples

**Figure 7:**
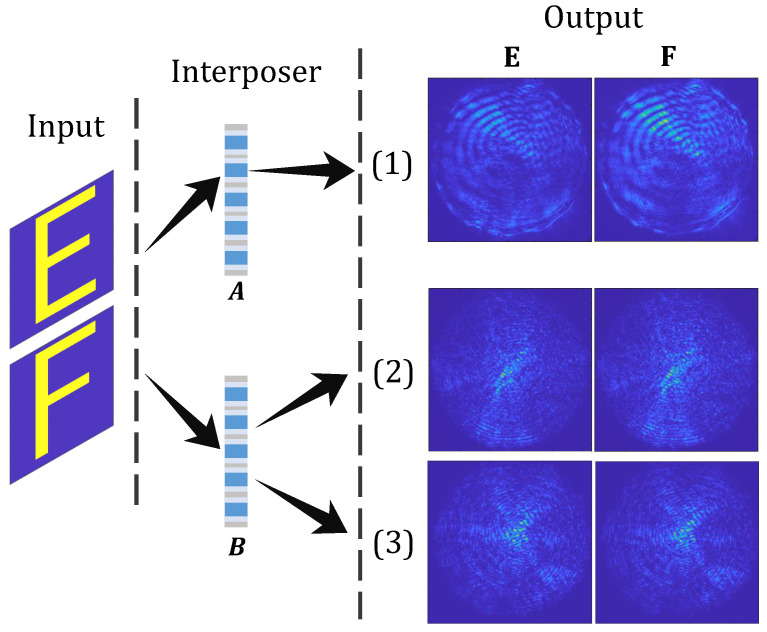
Output intensity distributions from different interposers, *A* and *B*, corresponding to projecting letters “E” and “F” at the input. (1) Response of one OTSV from interposer *A*; (2) response of one OTSV from interposer *B*; (3) response of another OTSV from interposer *B*.

As a third experiment, we compared the OTSV (from interposer A) used so far with two different OTSVs from interposer *B*. We again projected alphabetic letters on them. The corresponding output distributions from projecting “E” and “F” are shown in Figure 7. It can be seen that the output intensity distributions vary by switching the interposer and between the OTSVs of the same interposer. Correlating the output intensity distributions when “E” is projected at the input results in coefficients of 24% and 26% between the two interposers (OTSV (1) and (2) or (1) and (3), respectively; see Figure 7). When “F” is projected, the correlations are 18% and 26%. However, when the intensity within the different OTSVs of interposer *B* are correlated (OTSV (2) and (3), respectively), the correlation is 40% for “E” and 41% for “F” projected at the input. These results stress the uniqueness of a multimode channel created by an OTSV.

### 5.4. Recognition of the Speckle Patterns through a Deep Neural Network

**Figure 8:**
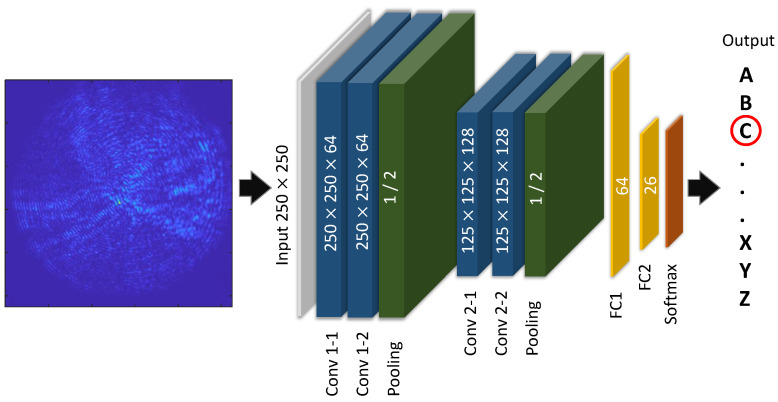
Structure of the VGG-type deep neural network. Here is an example of the input speckle pattern corresponding to the letter “C”.

Artificial intelligence, particularly deep learning algorithms, has garnered significant attention in a diverse range of research fields involving speckle pattern analysis [29], light manipulation [40], and medical diagnosis [41]. Based on previous work [42,43], it has been found that deep neural networks (DNNs) are good candidates for learning the relationship between the input and output of a complex medium [29,44]. Therefore, a DNN is introduced for distinguishing different speckle patterns, which seems hardly recognizable at first glance. The dataset used for the investigation consists of speckle images resulting from letter projections which are resized to 250×250 pixels as the DNN input with fixed dimensions. The prediction of the DNN is the label, i.e., the letter to which the speckle pattern corresponds. The structure of the DNN is inspired by the very deep convolutional network (VGGNet) [45] model. As shown in Figure 8, it has only six layers with learnable parameters and can be divided into three blocks. The first block consists of two convolutional layers and one max pooling layer. The convolutional layer is used to extract the features in the speckle pattern, and the max pooling layer is used for reducing the size of the channels and the learnable parameters. This is why the second block has the same structure as the first block, but the feature map size is halved. The convolutional layers in all of these networks have a kernel size of 3 × 3. The first block has 64 channels, which is then doubled to 128 channels in the second block. Two fully connected layers follow and can integrate all of the previously collected feature information. The output sizes of these two layers are 64 and 26, respectively. Finally, a softmax layer will predict which letter the input is most likely to be.

The DNN has been implemented in MATLAB, and the training is performed on a desktop computer with Intel Core i5-9500. Adam is chosen as the optimization algorithm to update the value of learnable parameters. The initial learning rate is set to 0.001 and is reduced by a factor of two every 20 epochs. After 60 iterations, the DNN eventually converged. The whole training process took about 8 min. With the 532 nm wavelength used in this experiment, each OTSV supports over 13,000 modes. Unlike long-distance optical fibers, the transmission characteristics of an OTSV are temporally stable due to its embedded structure within an interposer and its short length of only 380 μm [29]. Thus, after training, the DNN is able to classify speckle patterns for the same input. In the testing phase, the neural network predicted the labels of all speckle patterns with an accuracy value of 100%. Even in the case of “O” and “Q”, where the correlation coefficient has a value of 97%, the DNN can predict accurately. This result shows that DNNs can effectively discriminate complex speckle patterns. It should be noted that the DNN works as a classifier for fixed data in our investigation; the performance of the DNN on other types of data or overfitting is not investigated. This is attributed to the temporal stability of the OTSVs. In our study, we have observed that the output remains unchanged for the same input over several weeks. Signal power loss measurements were carried out one year apart, showing no deterioration in the OTSV’s performance [28]. This characteristic makes the DNN an excellent choice, as it exhibits consistent and stable predictions for known data. However, as a data-driven approach, the performance of the DNN relies heavily on the quality of the data, specifically the intensity images used in this study. Hence, it is crucial to minimize the presence of irrelevant light during the measurement process. Additionally, the unique transmission properties of each OTSV due to manufacturing tolerances pose a challenge for training a single DNN that can accurately handle all OTSVs. Therefore, a parallel speckle pattern recognition approach utilizing multiple DNNs is necessary for one interposer to enhance the transmission capacity.

## 6. Conclusions

The physical layer security approach for interposers was studied for the first time. Our experimental results demonstrate that multimode optical interconnects on the silicon interposer exhibit complex and unique transmission characteristics, although it has an interconnect length of only 380 μm. Their linear and temporally robust channels make the interposer naturally feasible for secure data transmission using physical layer security. We have shown that output intensity responses can be discriminated against by a DNN with 100% accuracy, even if they are highly correlated. These results show the possibility of utilizing structured light and deep learning techniques for secure optical communication. Compared to commonly used mathematical encryption methods at the application layer, physical layer security provides a higher level of protection by completely blocking access to data for eavesdroppers. Even with advancements in quantum computing and increased computing power, physical layer security remains effective in ensuring data security. The current primary challenges in employing physical layer security on interposers are the efficient processing of image information and high-speed opto-electronic signal conversion. These challenges can potentially be overcome through various techniques, including GPU acceleration, FPGA implementation, or the utilization of diffractive deep neural networks [46] and a combination of high-speed VCSEL array and multimode waveguides. Although the manufacturing technology for developing such an interposer is still in its early stages, the investigations on multimode optical interconnects on it showcase their great potential for high-speed communication using space division multiplexing and confidential optical data transmission toward secure 3D chip architectures.

## Data Availability

Not applicable.
